# Percutaneous vacuum-assisted tumor thrombectomy using angiovac and penumbra lightning 12 aspiration systems

**DOI:** 10.1016/j.radcr.2025.01.058

**Published:** 2025-02-04

**Authors:** Murtaza Jafri, Brittany Stojak, Owen Mooney, Andrew Macdiarmid, Ian W. Gibson, Surinder Dhaliwal

**Affiliations:** a Faculty of Medicine, University of Manitoba, Manitoba, Canada; b Department of Radiology, University of Manitoba, Winnipeg, Manitoba, Canada; c Department of Internal Medicine, Section of Critical Care, University of Manitoba, Winnipeg, Manitoba, Canada; d Department of Internal Medicine, University of Manitoba, Winnipeg, Manitoba, Canada; e Department of Pathology, Associate Professor Pathology, University of Manitoba, Winnipeg, Manitoba, Canada

**Keywords:** Mechanical thrombectomy, AngioVac, Penumbra indigo lightning device, Tumor thrombus, Intracardiac thrombi, Synovial sarcoma

## Abstract

A 19-year-old female presented with a 2-month history of abdominal discomfort and palpable abdominal mass; a computer tomography scan revealed a large retroperitoneal mass as well as high suspicion of thrombus in her inferior vena cava. Right kidney core biopsy showed diagnostic features of synovial sarcoma. While awaiting workup for her mass she was placed on anticoagulants. However, she re-presented to hospital 3 weeks later complaining of 3 days of shortness of breath. A repeat computer tomography scan revealed extensive thrombus burden with tumor thrombus involving the inferior vena cava, a large right atrial intracardiac thrombus, multiple segmental right pulmonary emboli, and a large embolus causing total occlusion of the left main pulmonary artery with findings suggestive of associated lung infarction. The patient's disease was not amenable to surgical resection and thrombolytic therapy was contraindicated due to associated intratumor hemorrhage, thus prompting consideration of thrombectomy under emergency approval from Health Canada. The patient underwent an overall successful total aspiration thrombectomy of the intracardiac tumor thrombus with subsequent resolution of her right heart strain, using the extracorporeal AngioVac aspiration system and partial aspiration of the extensive pulmonary emboli using the Penumbra Indigo Lightning 12 system. She was transferred to the intensive care unit and extubated 2 days later and was discharged from hospital shortly after without supplemental oxygen.

## Introduction

Synovial sarcomas are a rare subgroup of malignant tumours that make up approximately 5%-10% of all soft tissue sarcomas [1]. They arise from mesenchymal tissues and are thought to result from transformation and translocation of SYT gene (SS18) with SSX gene (SSX1/2/4) with the resultant protein disrupting normal cell regulation and proliferation of mesenchymal cells [2]. The disease commonly affects deep soft tissues adjacent to joints in the extremities, however, rarely can affect abdominal viscera and chest wall. The disease is a slowly enlarging soft tissue mass, possibly over years, with the mean onset between 15 and 40 years [1]. Synovial sarcomas commonly invade surrounding structures and vasculature leading to thrombus. While the incidence is not well studied specifically for synovial sarcomas, the incidence rate for thromboembolic events were found in approximately 7.9% of cases of sarcoma [3].

## Case presentation

**Fig. 1:**
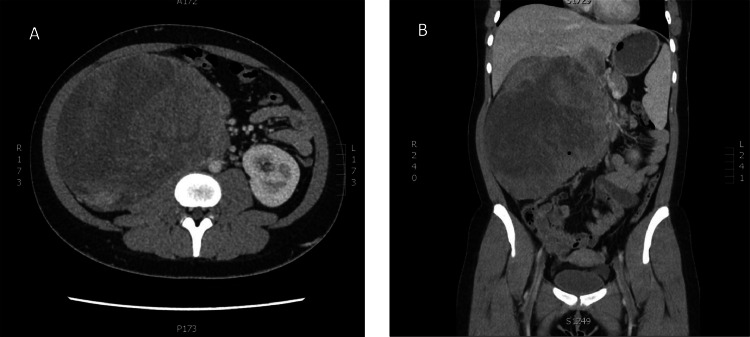
Axial CT showing (a) large tumor arising within the right retroperitoneum completely replacing the right kidney with tumor mass infiltrating the inferior vena cava. Coronal CT showing (b) the large retroperitoneal mass with thrombus extending into the proximal and distal IVC and right hepatic vein.

A previously healthy 19-year-old female presenting to the emergency room with a 2-month history of an enlarging, palpable right sided abdominal mass underwent a computed tomography (CT) scan as part of her diagnostic work up. The study showed a large, 20.6cm x 18.6cm x 16.4cm (craniocaudal x transverse x anterior-posterior) retroperitoneal mass with associated thrombus in the inferior vena cava (IVC) (Fig. 1). The patient was subsequently started on anticoagulation therapy with rivaroxaban 10mg PO daily while awaiting further work up.

Approximately 3 weeks after her initial CT scan, she presented to the emergency department with a 3-day history of acute onset dyspnea, pleuritic chest pain and hypoxemia. Subsequently, her rivaroxaban was held in anticipation of a biopsy and was not taken on the day of her presentation. Of note, her symptoms began 2 days prior to her anticoagulation being held.

**Fig. 2:**
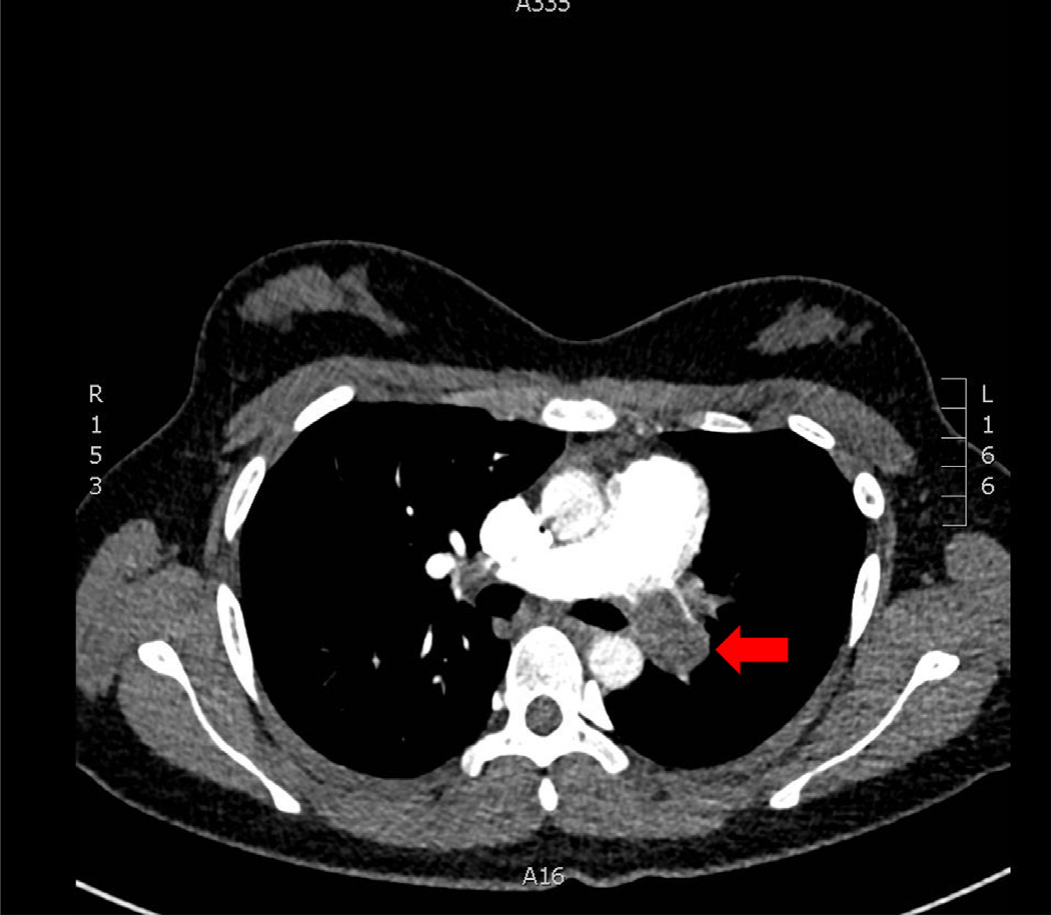
Chest computed tomography pulmonary angiogram (CTPA) showing total occlusion of the left pulmonary artery with signs of pulmonary infarction.

CT imaging revealed near total occlusion of the left pulmonary arterial tree with several small segmental right sided pulmonary emboli (PE) and a 3.0×2.0cm intracardiac, right atrial thrombus with concomitant evidence for right heart strain (Fig. 1b, Fig. 2). She was then admitted to hospital and started on a therapeutic heparin infusion in an attempt to mitigate right heart strain complications. During the same admission, she underwent an ultrasound-guided biopsy of the lesion. Ultrasound was chosen as the modality for the biopsy as the mass was a solid vascular retroperitoneal target which could be biopsied with real-time guidance to avoid high risk vasculature structures not well appreciated on CT. However, despite these precautions, complications of abdominal pain and hemodynamic instability with a 23g/L decrease in hemoglobin on postprocedure day 1 occurred. Repeat CT showed hemorrhage into the tumor with no identifiable arterial source. She was successfully managed conservatively with intravenous fluids alone.

**Fig. 3:**
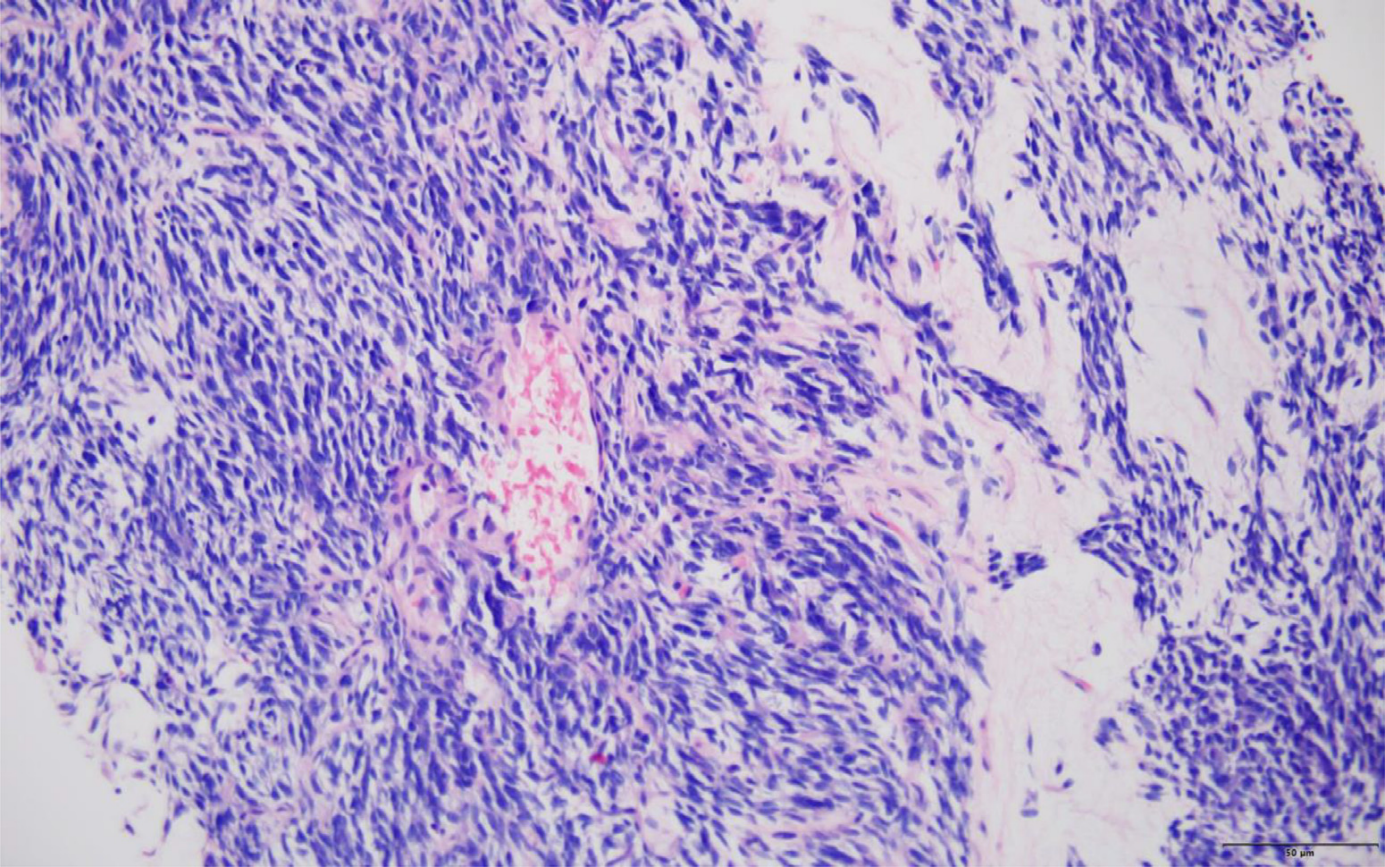
Right renal mass core biopsy showing cellular spindle cell tumor typical of synovial sarcoma. H&E stain, x200.

The right kidney mass core biopsy showed a cellular spindle cell neoplasm with fascicular architecture, focal myxoid stroma and mitotic activity (Fig. 3). Fluorescence in situ hybridization (FISH) showed tumor cells positive for SYT gene rearrangement, consistent with X;18 chromosomal translocation, diagnostic for synovial sarcoma.

**Fig. 4:**
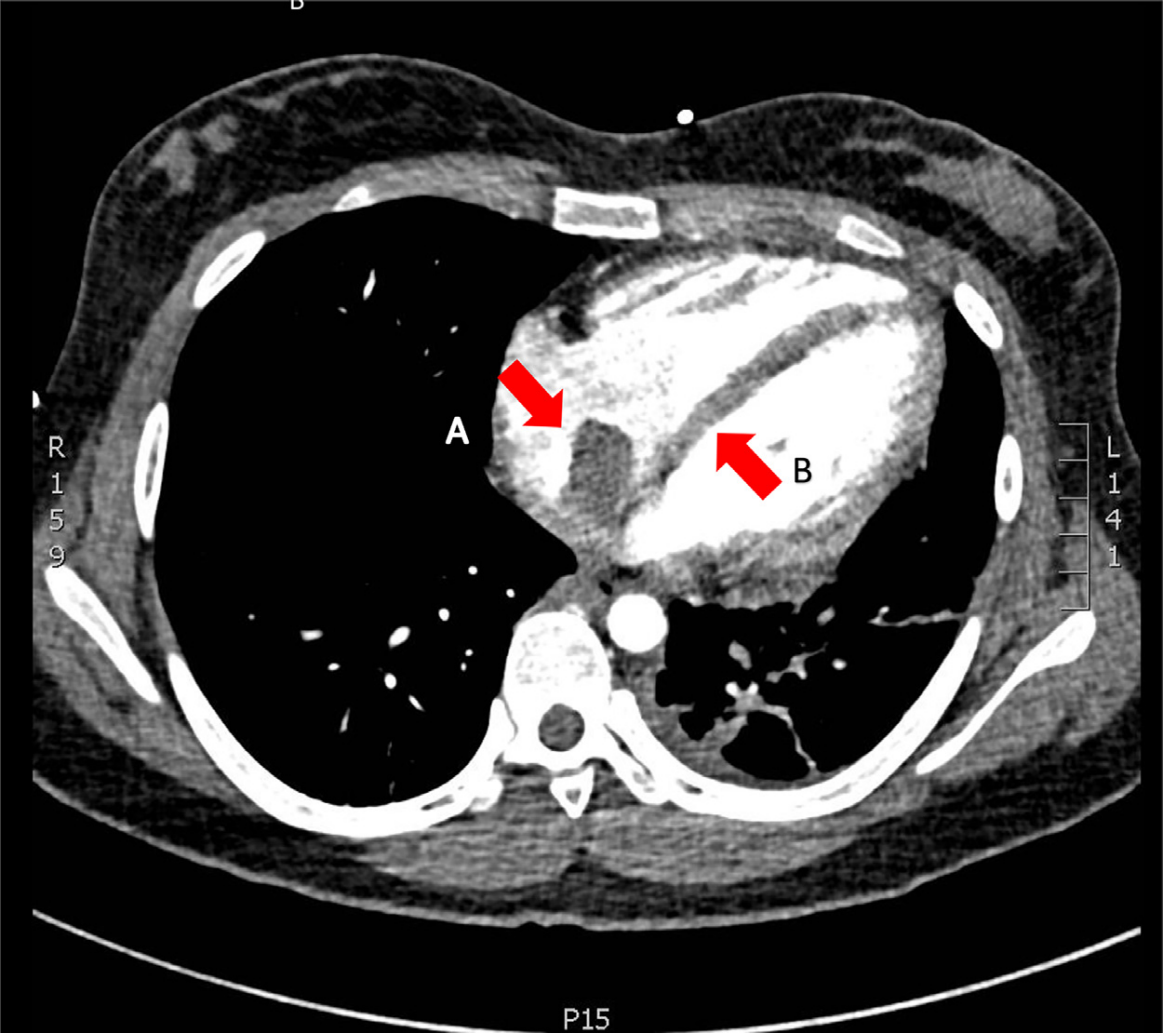
Chest computed tomography pulmonary angiogram (CTPA) showing a large intracardiac (a), clot-in-transit is seen within the right atrium with associated CT findings of right heart stain (b), note the flattening of the interventricular septum. *Not shown are extensive right-sided segmental pulmonary emboli.*

Given the extensive thrombus burden, the decision to resume anticoagulation with a therapeutic heparin infusion was made despite the intratumor hemorrhage. Despite ongoing anticoagulation, she experienced worsening pleuritic chest pain and dyspnea with increasing oxygen requirements. A CT pulmonary angiogram (CTPA) was performed to evaluate her worsening clinical symptoms. Results of the study suggested acute worsening of her right sided PE burden, largely unchanged right atrial thrombus, and ongoing total occlusion of the left pulmonary arterial circulation with new radiographic findings suggestive of left lung infarcts (Fig. 4).

Multidisciplinary discussions regarding best management occurred in collaboration with cardiac anesthesia, cardiac surgery, cardiology, radiology, internists, and perfusionists. The patient's thrombus burden and tumor properties were not amenable to open thrombectomy, and her intratumor hemorrhage was a contraindication to classical thrombolytic therapy. After extensive discussion with the patient, family and colleagues, percutaneous aspiration thrombectomy was pursued and its use in this patient with synovial sarcoma and extensive tumor thrombus burden is described.

## Procedure

**Fig. 5:**
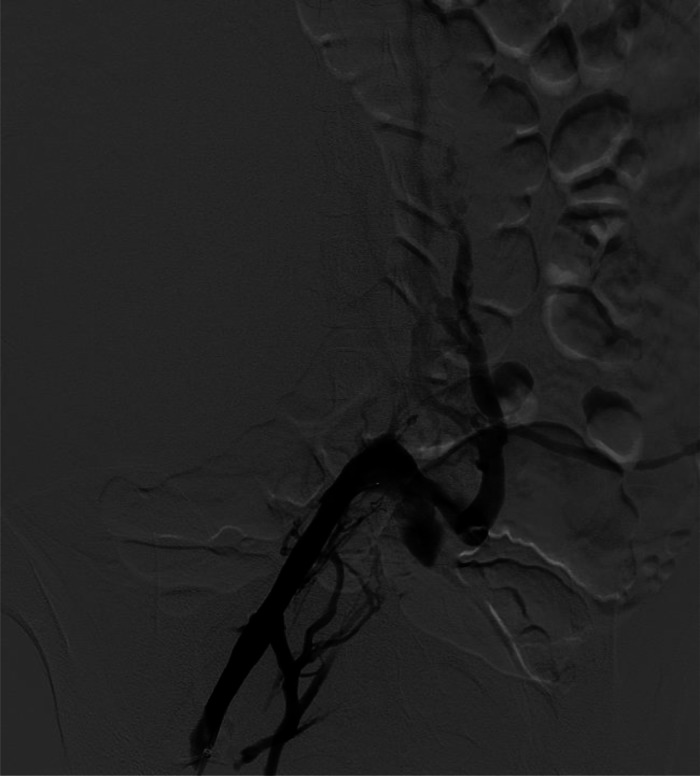
Intraoperative iliac venogram confirming occlusion of the inferior vena cava and extensive collaterals.

The procedure was performed under general anesthesia, with 2% Xylocaine used for local anesthetic. Percutaneous access was gained via ultrasound guidance to bilateral common femoral veins and bilateral internal jugular veins. The right common femoral vein access was not well suited for large cannula despite multiple tract dilators; this was converted to a small cut down. Intraprocedural iliac venogram confirmed extensive collaterals into the retroperitoneum and occlusion of the inferior vena cava (IVC) (Fig. 5). Direct visualization of the right atrium with transesophageal echocardiogram (TEE) and angiography was used to confirm thrombus location as well as cannula placement and guidance. A mid-esophageal 4 chamber, bicaval and several other TEE views were employed to visualize the thrombus within the right atrium and guide direct cannula placement. Aspiration of the right atrial thrombus in its entirety was performed using the AngioVac venous cannula System. The IVC was not explored as angiography revealed extensive tumor burden with integration with vessel wall and total occlusion of the IVC with extensive collateral vessels and the risks of massive hemorrhage outweighed any potential benefit (Fig. 5). Aspirated contents from the right atrium were sent to pathology. Surgical clips in the right groin were placed and the large infusion cannulas were removed with appropriate hemostasis achieved.

**Fig. 6:**
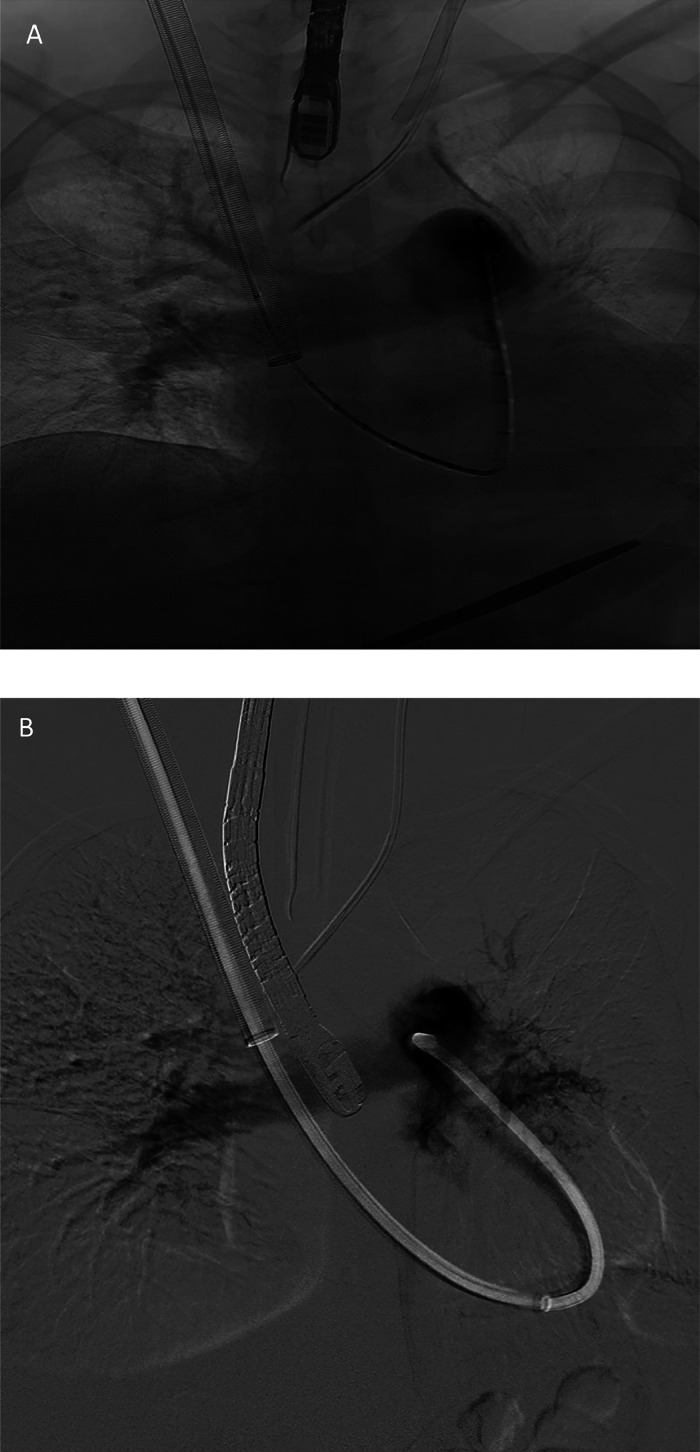
Intraoperative pulmonary angiograms showing the **(a)** preprocedure and **(b)** postprocedure left pulmonary flow.

Using the right internal jugular venous access, Penumbra Indigo Lightning 12 system was used to partially aspirate the left pulmonary artery with only modest improvement to blood flow as shown on angiogram (Fig. 6). Full removal was technically limited by poor visualization combined with the inability to confirm thrombus integration with vessel wall by TEE or rotational angiography coupled with known integration of thrombus with the IVC from the prior venogram (Fig. 5). In addition, large catheter size technically limited maneuverability within the pulmonary vasculature increasing the difficulty of extraction. The decision to limit harm was made and resulted in partial aspiration of pulmonary emboli. Aspirated clot and/or soft tissue from the left pulmonary artery were sent to pathology.

**Fig. 7:**
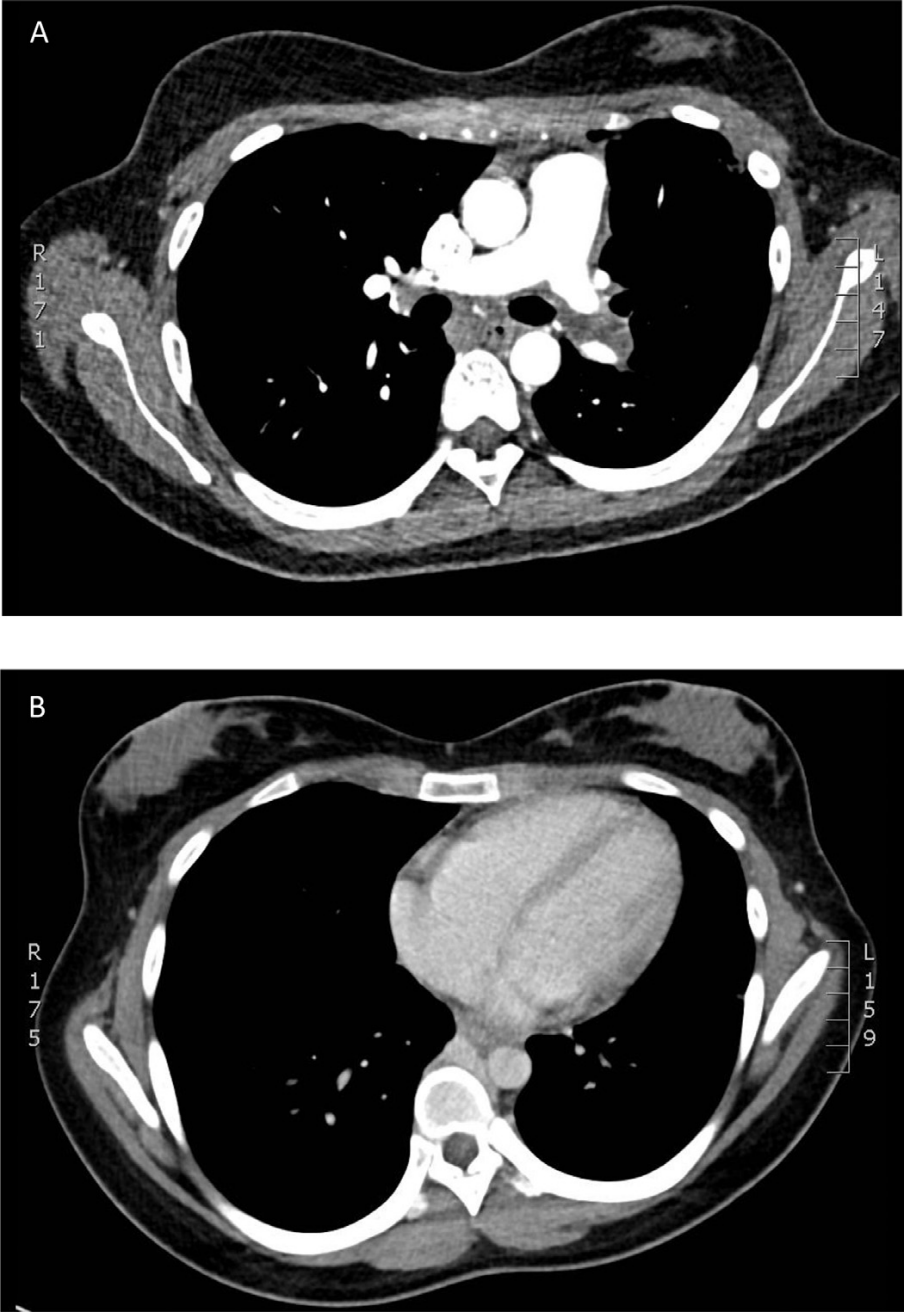
Follow-up computed tomography pulmonary angiogram (CTPA) showing **(a)** some improvement to the left pulmonary arterial flow and **(b)** right atrium free of thrombus and resolution of the radiographic findings of right heart strain.

**Fig. 8:**
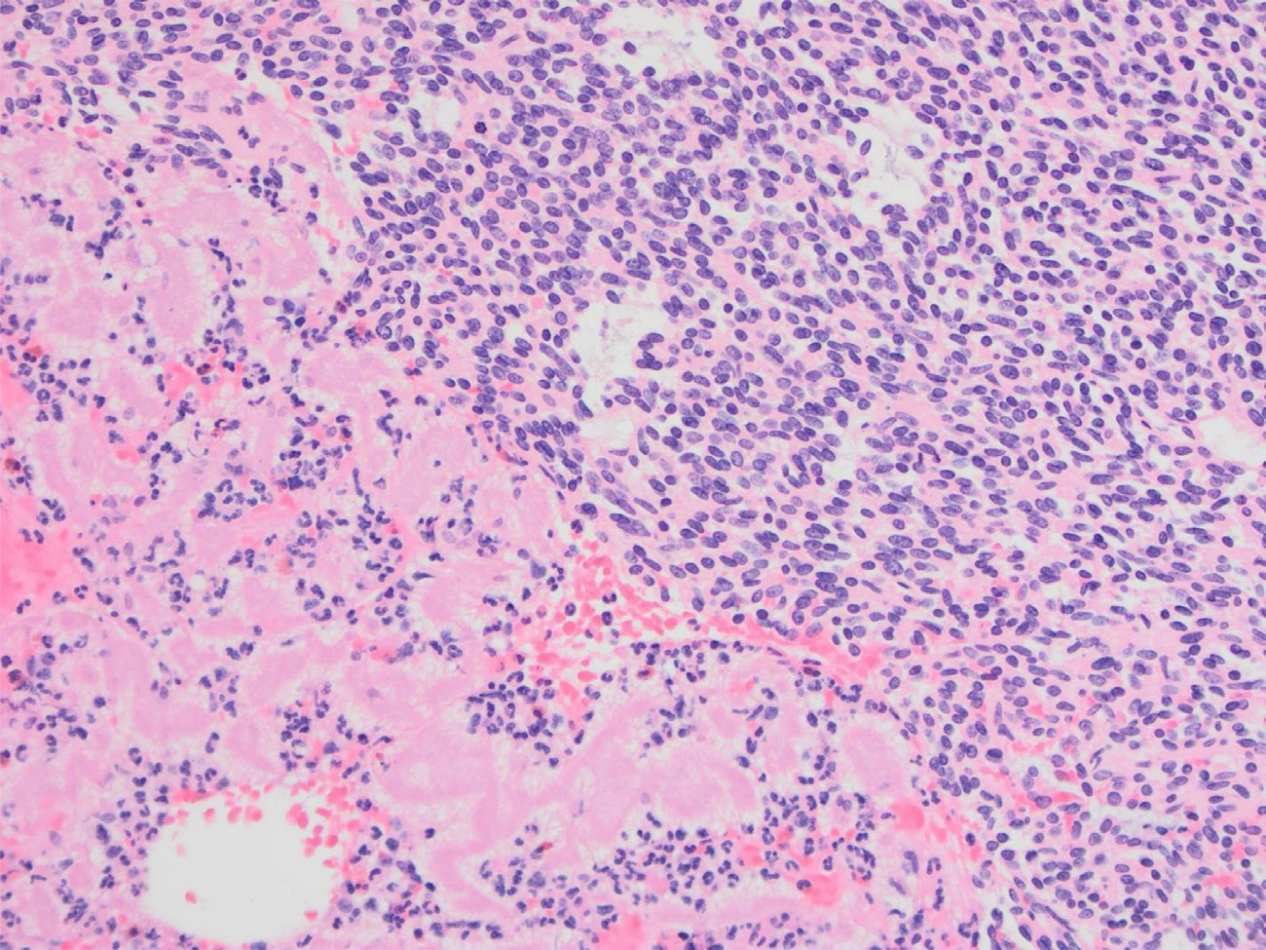
Thrombectomy specimen showing spindle cell tumor typical of synovial sarcoma with associated thrombus composed of fibrin, red blood cells and inflammatory cells. H&E stain, 200x.

The patient received intraprocedural heparin infusion with protamine reversal at completion. The patient tolerated the procedure well with hemodynamic stability intraoperatively as well as postoperatively without complications and was transferred to the ICU in stable condition. Follow-up computed tomography pulmonary angiogram (CTPA) showed some improvement to the left pulmonary arterial flow and right atrium free of thrombus and resolution of the radiographic findings of right heart strain (Fig. 7). The patient had significant improvement in her pleuritic chest pain, dyspnea and was able to come off oxygen support. Final pathology confirmed metastatic synovial sarcoma thrombus in all samples (Fig. 8).

## Discussion

Right heart thrombi are associated with high rates of cardiac arrest and are an independent predictor of mortality in untreated patients [4,5]. In those with pulmonary embolism and intracardiac thrombus, the risk of hemodynamic instability and death is even higher, with some estimates as high as 24%-29% in those treated with heparin alone and 11% in those treated with thrombolysis [6,7]. Differentiating thrombi as venous/bland vs tumor thrombus is challenging and has significant implications for prognosis and treatment options [8]. Tumor thrombus refers to intravascular tumor extension, most commonly into a vein. It can be seen in a wide variety of malignancies such as renal cell carcinoma, Wilm's tumor, adrenal cortical carcinoma, and hepatocellular carcinoma [8]. Many other tumors, both benign and malignant, may develop tumor thrombus, although less frequently. The presence of tumor thrombus significantly worsens prognosis and often has implications on treatment approach [8]. For example, an IVC filter should be avoided in the setting of tumor thrombus as it may integrate with the filter, making removal of both significantly more challenging [9]. Tumor thrombus often alters the surgical approach or may be a contraindication to surgical resection or transplantation as is the case in liver transplant for hepatocellular carcinoma [10]. Additionally, the presence of bland thrombus alone or in addition to tumor thrombus generally warrants consideration of anticoagulation therapy [9].

The mainstay treatment of large intracardiac or intravascular thrombus has historically centered on surgical embolectomy and thrombolytic therapy. However, studies such as the SEATTLE II trial have demonstrated that thrombolytic agents were associated with a 10% rate of major bleeding complications, defined by the GUSTO (Global Use of Strategies to Open Occluded Coronary Arteries) severe/life-threatening and GUSTO moderate criteria for bleeding event [11,12]. GUSTO severe/life-threatening bleeding is defined as either intracranial hemorrhage or bleeding resulting in hemodynamic compromise necessitating intervention and GUSTO moderate being defined as bleeding requiring transfusion, but not resulting in hemodynamic compromise [12]. Patients with active intratumor hemorrhage are poor candidates for these interventions, however, this subset of patients may benefit from percutaneous aspiration thrombectomy.

Multiple aspiration devices exist on the market, the AngioVac venous cannula system (Angio Dynamics, Lathan, New York) is 1 such device. Briefly, venous access is obtained with a large bore 22 Fr catheter through the internal jugular and femoral veins. The tip of the AngioVac cannula opens in such a way to create a flow vortex, pulling the thrombotic material in. The contents are filtered extracorporeally before being reinfused via bolus chase by a centrifugal pump console through the femoral access. This system was chosen in our case as the patient was anemic secondary to blood extravasation into her tumor, in addition to a nonfixed thrombus target. The ability to perform continuous aspiration of a mobile atrial thrombus provided greater flexibility peri‑procedurally while maintaining hemodynamic stability by delivering constant reperfusion without the need for transfusion. Another aspiration system called the Penumbra Indigo Lightning 12 system (Penumbra, Almeda, California) was chosen for the extensive pulmonary arterial thrombus as it possesses a large 12F reperfusion catheter with an angled tip and deployable separator wire that serves to mechanically disrupt thrombotic material, potentially allowing for extraction of larger volume clots such as in our case. In addition to being one of the only aspirations systems available to the hospital and having to seek emergency approval from Health Canada, having colleagues with expertise who have used the devices in the past with technical success, as well as evidence of their success made the combination of systems most appropriate for the case.

For the majority of patients, systemic anticoagulation is still considered the mainstay treatment for massive pulmonary emboli and intracardiac bland or mixed thrombus. The role of anticoagulation is limited in pure tumor thrombus**.** In a single-center retrospective cohort study where 86 patients with tumor thrombus were identified, patients receiving anticoagulation still developed venous thromboembolism in addition to an increase in major bleeding events compared to those without anticoagulation [13]. Therefore, it is not unreasonable to suggest that those who fail anticoagulation, thrombolysis or embolectomy may be viable options. However, many patients have contraindications to thrombolysis with up to 40% of patients being poor candidates [14]. In this population, interventional treatments, such as percutaneous aspiration thrombectomy, should be considered. A variety of catheter-based thrombectomy procedures exist, functioning through aspiration or fragmentation. Historically, these thrombectomy methods necessitated concomitant thrombolytic therapy, limiting their use in certain subsets of patients. This report described the use of the AngioVac venous cannula system and the Penumbra Indigo Lightning 12 system for thrombus extraction without concurrent thrombolytic therapy. The Penumbra Indigo Lightning 12 system is a 12F catheter with circumferential sweep and large lumen optimized for maximal thrombus extraction. The Evaluating the Safety and Efficacy of the Indigo Aspiration System in Acute Pulmonary Embolism (EXTRACT-PE) study is a 119-patient, prospective, multicenter study that showed significant reduction in the right ventricle to left ventricle ratio, a surrogate for right heart strain, when using the Indigo aspiration system for pulmonary emboli extraction [15].

The AngioVac catheter is a large-bore 22 Fr aspiration catheter with a balloon-actuated tip that uses a perfusion circuit to allow for forceful en bloc aspiration of clot or intravascular thrombus without the need for concomitant thrombolysis. The Angio Dynamics Medical AngioVac Cannula is Food and Drug administration (FDA) approved for use as a venous drainage cannula during extracorporeal bypass for up to 6 h. Previous small-scale studies have shown the most common complication to be a postprocedure drop in hemoglobin suspected to be related to blood pooling within the circuit system itself [16]. A 16-patient retrospective study showed no patients with overt bleeding or pulmonary hemorrhage, with all of them remaining hemodynamically stable during the procedure [16]. In addition, the Registry of AngioVac Procedures in Detail (RAPID) study collected data from 234 patients who received interventions using the AngioVac for caval thromboembolic, right heart masses, catheter-related thrombi, and pulmonary emboli overall showing efficacy and safety for its use in vascular thrombi and cardiac masses [17]. In patients with thrombus, techniques involving the removal of 70%-100% of the thrombus with the AngioVac system for cardiac thrombi were achieved in 58.5% of cases demonstrating their efficacy in complex cases without options for alternative treatments [17]. However, there was insufficient sample size within the pulmonary embolism group to comment on safety and efficacy within this sub population [17].

Although the AngioVac system and the Penumbra Lightning 12 System may widen the scope of those eligible for percutaneous thrombectomy, its use is limited by several factors. Difficult extractions complicated by poor visualization or tumor thrombus limits the extent of our aspirated contents while also increasing the risk of morbidity to patients. Cost burdens of the AngioVac thrombectomy system must be considered and compared to more economical options for endovascular treatment of venous thrombi or more widely available medicalized treatments. In addition, costs associated with general anesthesia and a cardiac perfusionist are also factors likely limiting its use to tertiary cardiac centers. Costs can be considered in conjunction with the needs for thrombolytic therapy requiring intensive care monitoring for hemorrhaging risks with limited options for further treatment.

The large size and inflexibility are somewhat mitigated with increased development allowing for 22 Fr with either 20-degree to 180-degree angled tips or 18 Fr with 85-degree angled tips, permitting increased distal aspiration with adequate access in addition to increased maneuverability. Although mechanically advanced, use of the aspiration system is technically challenging, requiring expertise and experience to avoid risks and complications of the procedure.

Despite the limitation of case report designs, small sample size, a single center report, this study supplements a limited number of similar reports and studies detailing the specific indication and outcomes for the utility of the AngioVac Cannula and the Penumbra Lighting 12 systems within patients with contraindications to thrombolysis and concomitant tumor thrombus burden. More reports with appropriately controlled data are needed to assess the value of these aspirations systems on patient morbidity and mortality.

## Conclusion

Using the AngioVac aspiration system, a technically successful and complete intracardiac thrombus aspiration was achieved in a 19-year-old female with history of synovial sarcoma. Although limited by poor technical visualization, the Penumbra Indigo Lightning 12 system was partially successful in aspirating extensive smaller segmental pulmonary emboli and soft tissue aggregates pathologically consistent with synovial sarcoma. There were no postprocedural complications, and the patient was later discharged with symptomatic improvement. This technique may serve as an alternative treatment option for management of intracardiac and intravascular thrombus extraction, especially for patients who are not candidates for open thrombectomy or who have contraindications to thrombolytics. Technical success of tumor thrombus aspiration was achieved with a combination of appropriate radiographic visualization using real-time TEE and angiography providing visualization of feasibility and safety of thrombus removal, coupled with optimization of catheter size and maneuverability of devices. We have demonstrated technical success of complete extraction of right heart tumor thrombus in addition to partial extraction of pulmonary arterial tumor thrombus leading to increased support for these devices in well visualized tumor thrombus secondary to a sarcomatous origin.

## Patient consent

Informed consent for publication was obtained from patient.
